# Tracking Membrane Protein Dynamics in Real Time

**DOI:** 10.1007/s00232-020-00165-8

**Published:** 2021-01-07

**Authors:** Fredrik Orädd, Magnus Andersson

**Affiliations:** Department of Chemistry, Umeå University, Umeå, Sweden

**Keywords:** Membrane protein dynamics, MD simulation, X-ray solution scattering

## Abstract

**Abstract:**

Membrane proteins govern critical cellular processes and are central to human health and associated disease. Understanding of membrane protein function is obscured by the vast ranges of structural dynamics—both in the spatial and time regime—displayed in the protein and surrounding membrane. The membrane lipids have emerged as allosteric modulators of membrane protein function, which further adds to the complexity. In this review, we discuss several examples of membrane dependency. A particular focus is on how molecular dynamics (MD) simulation have aided to map membrane protein dynamics and how enhanced sampling methods can enable observing the otherwise inaccessible biological time scale. Also, time-resolved X-ray scattering in solution is highlighted as a powerful tool to track membrane protein dynamics, in particular when combined with MD simulation to identify transient intermediate states. Finally, we discuss future directions of how to further develop this promising approach to determine structural dynamics of both the protein and the surrounding lipids.

**Graphic Abstract:**

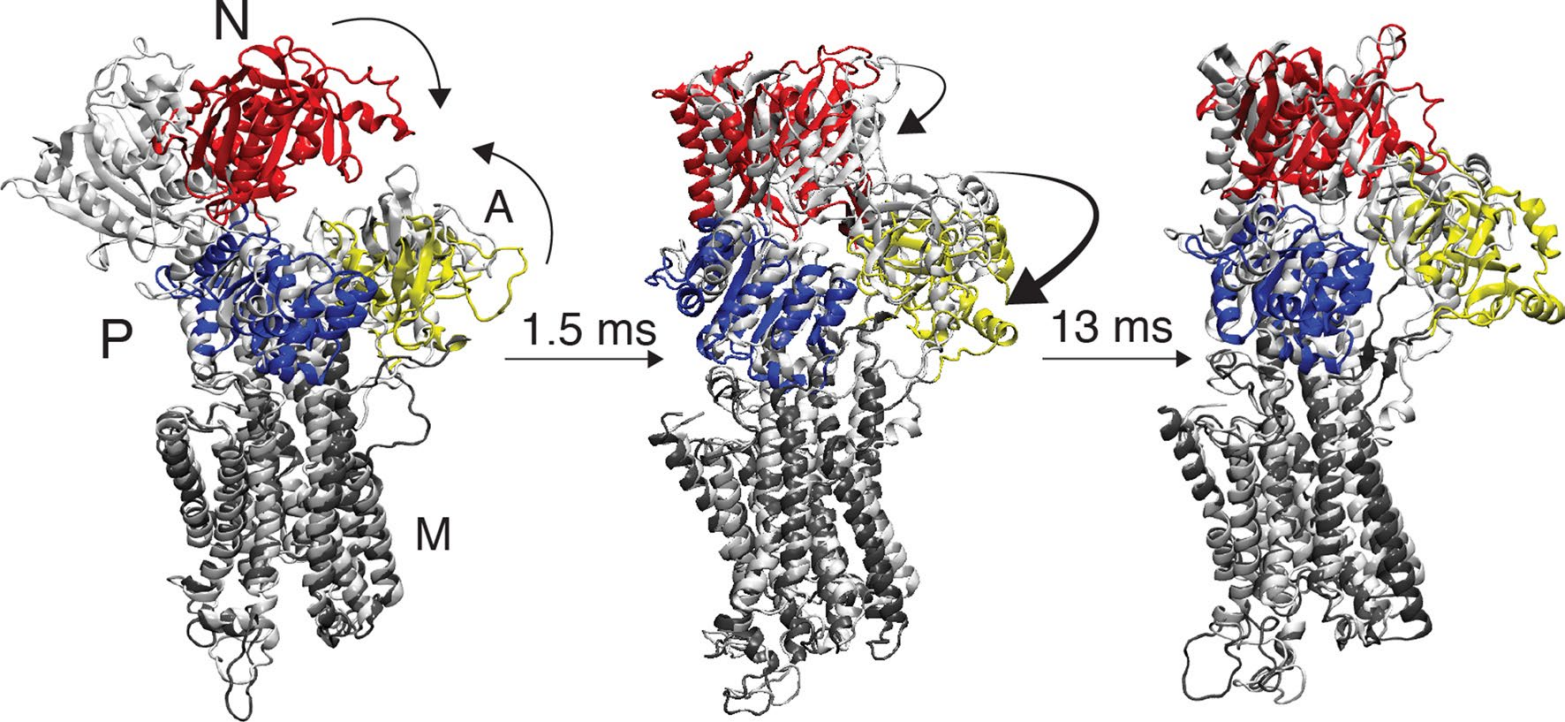

## The Membrane Protein Influencer

**Fig. 1:**
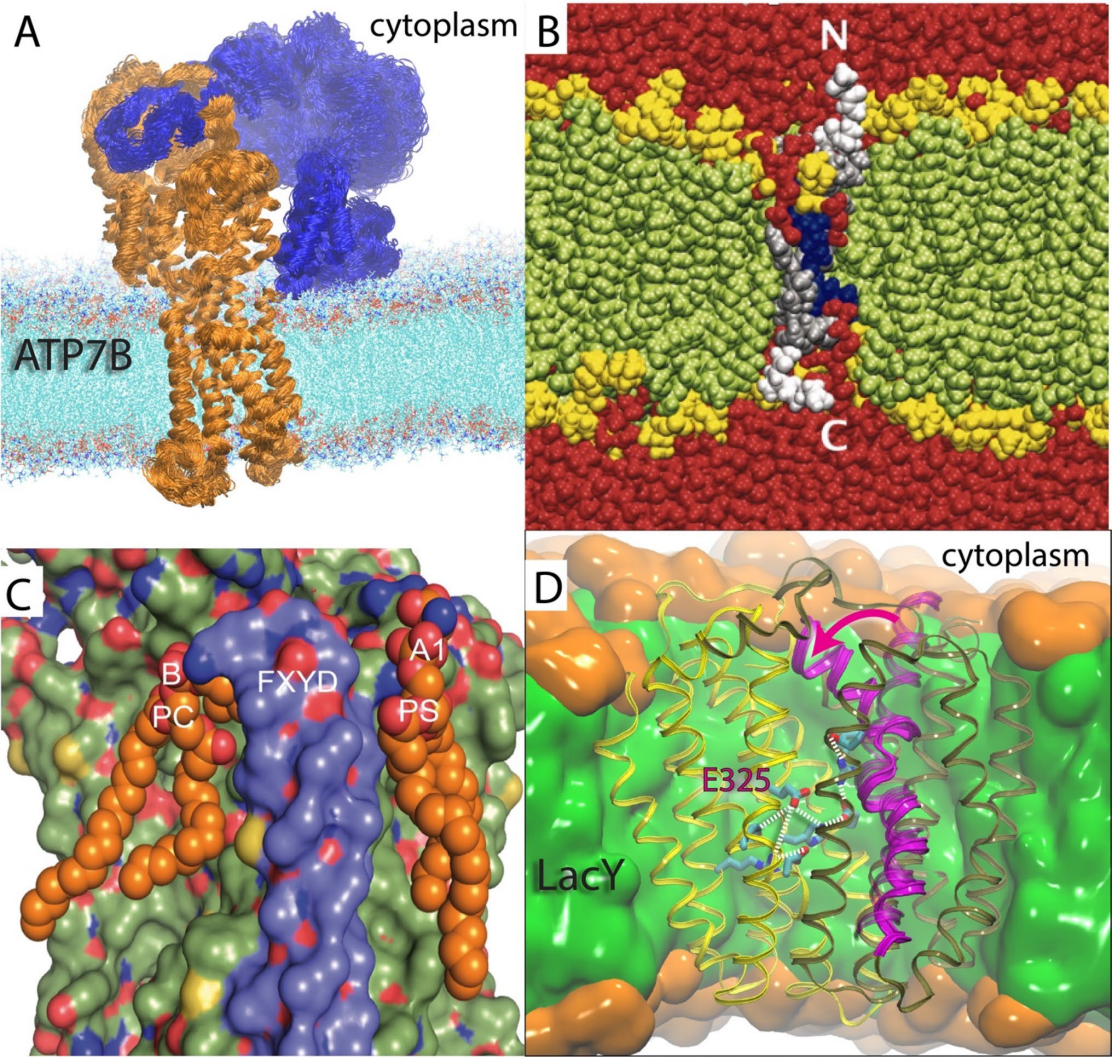
Membrane protein structural rearrangements and lipid regulation. **a** Homology model of the human Cu^+^-transporting P-type ATPase (ATP7B) (orange) with associated regulatory domain (blue) inserted into a lipid bilayer. Several simulated overlayed structures visualize thermal fluctuations displayed within a single state of the reaction. **b** A simulated S4 voltage-sensor peptide (silver with white GGPG flanks) showing bilayer distortion as the charged residues (blue) become solvated by lipid phosphates and water molecules (red). The lipid headgroups and tails are displayed in yellow and green, respectively. Adapted from Freites et al. (2005). **c** Lipid sites (A1 and B) hosting PC and PS lipids remodeled from a crystal structure at the cytoplasmic side of the Na^+^, K^+^ ATPase transporter. Adapted from Cornelius et al. (2015). **d** Lipid-dependent dynamics (magenta arrow and helix) and internal trigger (protonation state of residue E325) in the Lactose permease (LacY) transporter. The C-terminal and N-terminal domains are colored yellow and tan, respectively. The hydrophobic core of the membrane is colored green, and the polar headgroup region is depicted in orange. Adapted from (Andersson et al. 2012)

Membrane proteins execute critical cellular processes and enable—with incredible diversity and ingenuity—life as we know it. To appreciate the courage of a membrane protein researcher, it is illustrative to consider the typical spatiotemporal scale and operating conditions of such biological macromolecules. First, the lipid composition varies drastically in membranes across organelles, tissues, and organisms—and also in-between the bilayer leaflets (Harayama and Riezman 2018). In addition, the lipid bilayers display a wide range of structural dynamics from local structural rearrangements (Wiener and White 1992) to transient formation of lipidic micro-assemblies, referred to as lipid rafts (Simons and Sampaio 2011). Second, membrane proteins become inserted into its complex lipid environment by the translocon machinery—itself being a protein—in a process still holding lingering mysteries (Cymer et al. 2015). Third, membrane protein function depends on carefully orchestrated subtle structural rearrangements of amino acid side chains and large-scale conformational changes that span several orders of magnitude on the temporal scale (Fig. 1a). The emerging view is that membrane proteins have coevolved with the membrane lipids to optimize functionality (Lee 2004). Therefore, one of the intricate regulation machineries that control membrane protein activity are the physicochemical properties of the lipids in the surrounding membrane. The endeavor to understand membrane protein function—and associated disease—should therefore ideally monitor the reaction directly in the native membrane on the biological time scale.

Prime examples of the complex protein-lipid interplay are voltage-gated sodium, potassium, and calcium channels that govern nerve impulses. In these proteins, a central ion-conducting pore opens and closes in response to movements of associated voltage-sensing domains induced by changes in the electrical potential (Swartz 2008). The membrane in the vicinity of the voltage-sensing domains deforms locally and enables the formation of a water-filled crevice that hydrates critical acidic and basic amino acid residues, thus allowing them to keep the electrical charge—and hence the capability to respond to changes in the membrane potential (Krepkiy et al. 2009). The water hydration pattern around simulated isolated charge-carrying S4 helices or voltage-sensing domains was shown to depend upon the presence of lipid phosphates in the surrounding membrane (Sands and Sansom 2007; Freites et al. 2005; Andersson et al. 2011) (Fig. 1b), which provides a possible molecular explanation of the lipid head group requirements for optimal functioning of voltage-gated potassium channels (Schmidt et al. 2006; Ramu et al. 2006; Xu et al. 2008). In membranes of heart cells and neurons, polyunsaturated fatty acids (PUFAs) activate voltage-gated potassium channels via electrostatic interaction (Xu et al. 2008; Borjesson et al. 2008) with specific sites of interaction predicted by molecular dynamics (MD) simulation (Yazdi et al. 2016). In addition, phosphatidylinositol 4,5-bisphosphate (PIP_2_) anionic lipids also regulate voltage-gated potassium channels, in particular the subcategory KCNQ channels (Kruse et al. 2012; Duncan et al. 2020). Regulation includes promoting the coupling between the central ion-conducting pore and the peripheral voltage-sensing domains thus affecting voltage sensitivity (Zhou et al. 2013; Zaydman et al. 2013; Zhang et al. 2013; Kim et al. 2017), which has been located to a linker region connecting the two domains (Rodriguez-Menchaca et al. 2012). A crystal structure of an open-state channel resolved a phosphatidylglycerol (PG) lipid, which is also negatively charged, at the linker region (Long et al. 2007) and several simulations have observed a similar PIP_2_-interaction site (Kasimova et al. 2014; Kasimova et al. 2015). A second PIP_2_-interaction site has been proposed to display preference for the closed state of the channel and could therefore possibly be involved in channel deactivation (Zaydman et al. 2013; Zhang et al. 2013; Eckey et al. 2014). Indeed, MD simulations observed PIP_2_ migration between both sites (Chen et al. 2015). Finally, as an extreme case, PIP_2_ directly activates so-called inwardly-rectifying potassium channels (Huang et al. 1998).

Pentameric ligand-gated ion channels (pLGICs) bind neurotransmitters and govern fast synaptic transmission (Plested 2016). A common structural basis consists of a central ion-conducting pore forming in-between 4-helical membrane domains from the five subunits. Ligands bind to large extracellular subunits that transmit gating signals to the membrane pore region, which has been observed by e.g. series of crystal structures (Hu et al. 2018) and, at least partially, by MD simulation (Yoluk et al. 2015; Polovinkin et al. 2018; Guros et al. 2020; Damgen and Biggin 2020; Lev and Allen 2020). Several pLGICs show strict requirements for certain membrane lipids (Thompson and Baenziger 2020). Cryo-EM structures in nanodiscs composed of lipids known to disrupt functionality, showed significant structural change between agonist-bound and apo structures in the extracellular domains, but not in the membrane domains (Kumar et al. 2020), thus providing a structural explanation for the lipid dependency. In addition, direct binding has been observed of e.g. phosphatidylglycerol (PG) (2019) and phosphatidylethanolamine (PE) (Henault 2019) lipids and cholesterol (Henin et al. 2014; Brannigan et al. 2008;Zhu et al. 2018; Sharp et al. 2019), that modulates function.

G protein-coupled receptors (GPCRs) induce intracellular signaling in response to external binding of a wide range of stimuli, such as photons and small molecules (Pierce et al. 2002). Ligand binding mediates structural rearrangements in the seven-transmembrane protein that results in G protein dissociation and the intracellular response. Membrane-embedded cholesterol has been identified as critical to GPCR function (Oates and Watts 2011) with proposed effects on ligand binding (Pucadyil and Chattopadhyay 2004), stability (Zocher et al. 2012), and oligomerization (Chakraborty and Chattopadhyay 2015). Advances in GPCR structural biology have generated more than 70 unique receptor structures and signaling complexes (Wang et al. 2020; Congreve et al. 2020), which have resulted in significant insight into protein-lipid interactions by MD simulation approaches. For instance, several cholesterol-binding sites have been identified (Lee and Lyman 2012; Sengupta and Chattopadhyay 2012; Shan et al. 2012), and also have shown that binding alters the conformational state of the receptor (Manna et al. 2016). An observed decrease in the number of hydrogen bonds in the cholesterol-containing protein-lipid interface provides a possible molecular explanation to the enhanced conformational freedom in the presence of cholesterol (Ramirez-Anguita et al. 2018), and the affinities of cholesterol, ganglioside (GM3), and PIP_2_ lipids were shown to differ between different conformations of GPCR structures (Song et al. 2019). In addition, MD simulations have also shown that the degree of membrane disorder, as affected by lipid composition, facilitate oligomerization and that negatively charged lipids affect activation—but not oligomerization (Marino et al. 2016).

Other examples of membrane proteins with function that depends upon the membrane lipids are ATP-dependent P-type ATPases that transport ions, and also lipids, against their gradients. The Na^+^,K^+^ P-type ATPase maintains electrochemical gradients by ATP-dependent transport of three intracellular Na^+^ ions and two extracellular K^+^ ions across the membrane and thereby contributes to the membrane resting potential (Nyblom et al. 2013). Several sites of direct lipid-protein interaction have been identified in crystal structures (Cornelius et al. 2015) that have been shown to affect activation, stabilization, and inhibition of transport activity in detergent/lipid micelles (Habeck et al. 2017; Habeck et al. 2015) (Fig. 1c). Such lipid-protein interaction sites have also been identified in crystal structures of Ca^2+^ P-type ATPase proteins that govern e.g. muscle relaxation (Drachmann et al. 2014). These Ca^2+^ transporters, or SERCA proteins, have adapted to the thickness of sarcoendoplasmic reticulum membranes (Johannsson et al. 1981) and this adaptation has been assigned to specific steps in the reaction cycle, such as phosphorylation and dephosphorylation (Michelangeli et al. 1991). Due to the unfavorable thermodynamics involved in exposing water to hydrophobic amino acids in the membrane domain of the protein, any mismatching to the surrounding membrane can be expected to be minimized. Both the SERCA protein itself and the inner layer of the surrounding lipids was shown to adapt structurally to different thicknesses of the membrane (Sonntag et al. 2011). Such adaptation has also been shown to induce tilting of the SERCA protein in mixed lipid-detergent micelles (Norimatsu et al. 2017).

The archetypal member of the major facilitator superfamily, the lactose permease of *Escherichia coli* (LacY), which imports galactopyranoside sugar in a secondary active transport mechanism (Kaback 2015), requires phosphatidylethanolamine (PE) lipids for proper insertion and active transport (Bogdanov et al. 2002). MD simulations identified PE-interaction sites in LacY that were unable to accommodate phosphatidylcholine (PC) lipids, leading to a loss of observed local and global functional dynamics in PC lipid bilayers (Andersson et al. 2012) (Fig. 1d). In addition to such direct lipid-protein interactions, the general physicochemical properties of the membrane also affect LacY structure and function (Bogdanov et al. 2010). While folding, stability, and function of LacY all depend critically on the composition of the lipid bilayer, these properties show significant differences in their lipid dependencies (Findlay and Booth 2017). The LacY transporter is a cardinal example of the complexity of lipid regulation of membrane protein systems. To understand the molecular basis of how lipids can act as allosteric modulators, and how lipid composition can be engineered to curb membrane protein-associated disease, will take persistence, novel methodology, and courage.

## The Spatial- Vs. Temporal-Scale Dilemma

**Fig. 2:**
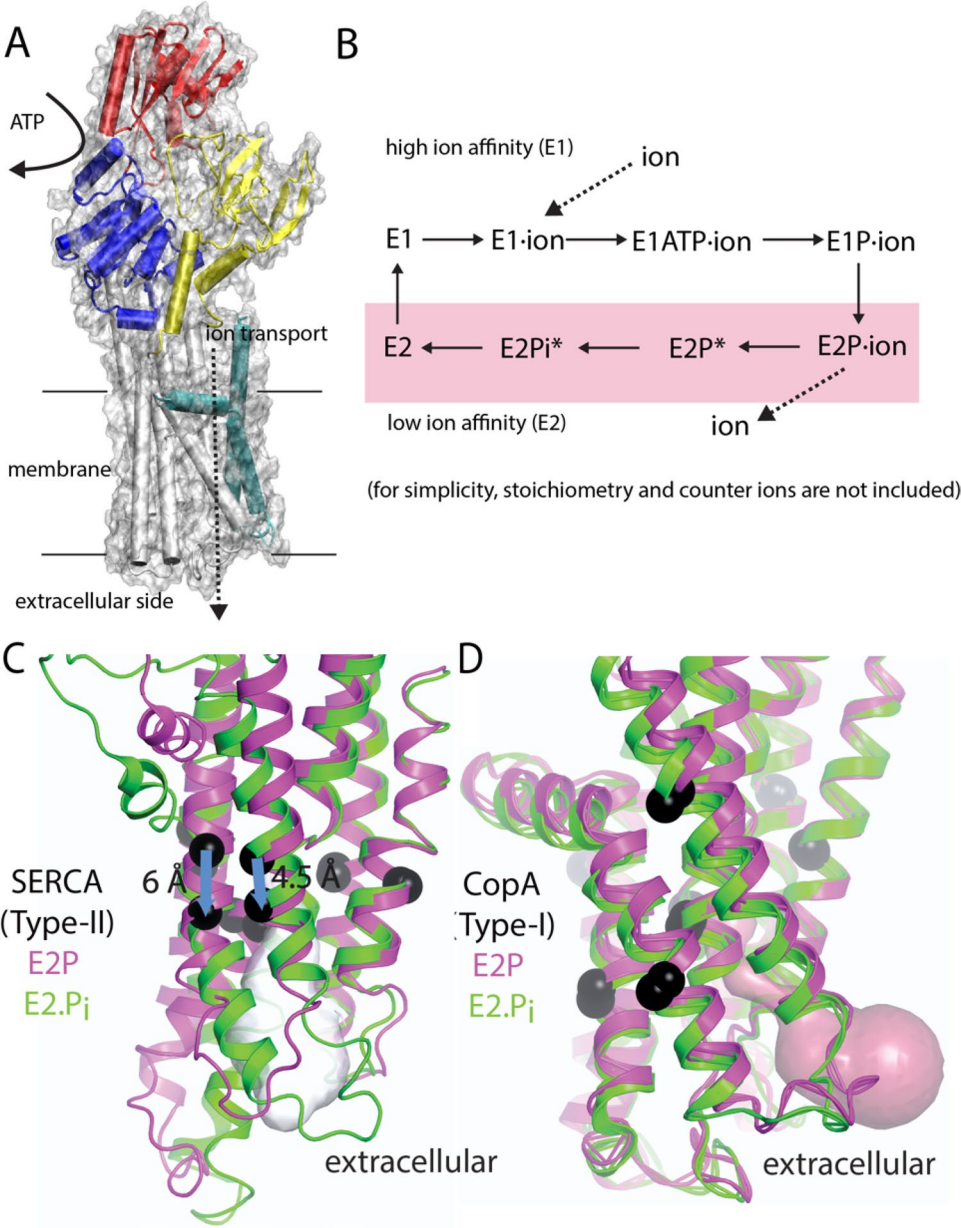
P-type ATPase structure, transport reaction, and subtype differences. **(a)** P-type ATPase architecture, exemplified by a bacterial Cu^+^-transporting ATPase (CopA), with nucleotide-binding (N), phosphorylation (P), and actuator (A) domains in red, blue, and yellow, respectively. The part of the membrane domain common to all P-type ATPases is shown in white and the CopA-specific helices in cyan. **(b)** P-type ATPase reaction scheme showing shifts in ion affinity (E1/E2) and accompanying phosphorylation events. Asterisks mark the existing Zn^2+^ and Cu^+^ ATPase crystal structures. A side-by-side comparison of the E2P (magenta)-to-E2Pi (green) transition of **(c)** SERCA and the **(d)** Cu^+^ ATPase highlights differences in structural dynamics in the transmembrane domain involved in ion release (black spheres correspond to similar Cα positions in each state). Adapted from Andersson et al. (2014)

With technical advances and development of novel enhanced sampling algorithms, MD simulation has emerged as a powerful tool for biophysical characterization of complex membrane protein systems (Enkavi et al. 2019). Even though unbiased, atomistic simulations cannot sample the biological time scale, i.e. quite frequently reaction cycles of tens-to-hundreds of milliseconds, certain critical aspects of the membrane protein reactions can be simulated at atomistic detail. For example, the membrane domain of P-type ATPases is attached to large protruding cytoplasmic domains in which the ATP hydrolysis takes place (Fig. 2a). These domains then undergo large-scale conformational changes that drive the transition from so-called E1 states that are open to the cytoplasm, to E2 states with ion-binding sites instead exposed to the extracellular side (Fig. 2b). While crystal structures of the sarcoendoplasmic reticulum calcium ATPase (SERCA) protein, trapped in different modes of action, have contributed enormously to understanding of the transport reaction (Dyla et al. 2019), biophysical characterization, including MD simulation, has added necessary details on the conformational dynamics involved in e.g. activation, inhibition, and regulation (Dyla et al. 2019; Aguayo-Ortiz and Espinoza-Fonseca 2020).

Several crystal structures have paved way for a better understanding of P-type ATPases that regulate cellular heavy-metal homeostasis (so-called Type-I ATPases) (Gourdon et al. 2011; Andersson et al. 2014; Wang et al. 2014), but dynamic processes such as entry and release of ions, hydration dynamics, and membrane partitioning remained elusive. Predictions from MD simulations of ion release enabled determination of an ion-release pathway in Cu^+^-transporting P-type ATPase (CopA) proteins (Andersson et al. 2014). By comparing to the Type-II SERCA transporter, it was clear that copper transport was associated with unique structural changes in the membrane domain (compare Fig. 2c and d). Interestingly, the ion-release mechanism in Zn^2+^-transporting P-type ATPase (ZntA) proteins, on the other hand, was reminiscent of the prototype SERCA reaction (Wang et al. 2014), which highlights mechanistic variations within the heavy metal-transporting subfamily. Due to the lack of crystal structures trapped in ion-binding states, how heavy-metal ions enter and bind to the internal transport sites remain unknown. A combined modeling-simulation approach predicted membrane partitioning of a protein-associated platform for ion-delivering chaperones and a putative ion-entry mechanism in Cu^+^-transporting (CopA) P-type ATPases (Gronberg et al. 2016). Hence, atomistic MD simulations can clearly provide critical insight into dynamical processes in membrane proteins that are difficult to assess with experimental methods, but the method is limited by the time scales that can be sampled, which frequently correspond only to a fraction of the full reaction.

**Fig. 3:**
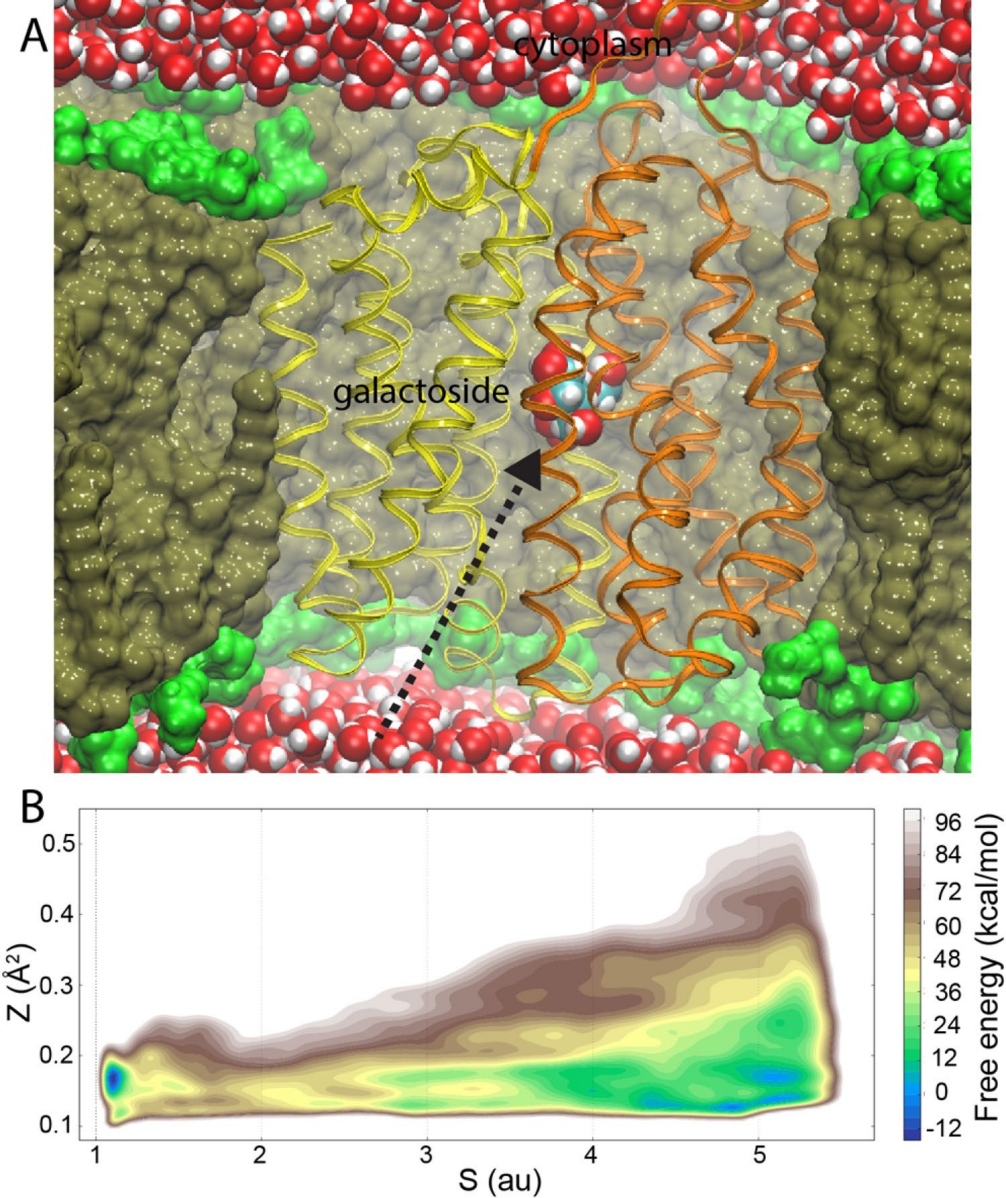
Sugar-binding energetics determined by metadynamics-enhanced sampling. **a** LacY crystal structure open towards the periplasm inserted into a lipid bilayer with hydrophobic core and polar headgroups in brown and green, respectively, with water molecules solvating both sides of the membrane. Extended, unbiased MD simulations identified a sugar-uptake pathway from the periplasm (dashed arrow). **b** Metadynamics simulations using the location of the uptake pathway (Z and S) as collective variables determined the free energy associated with sugar uptake and binding with excellent agreement to experimental data. Adapted from Kimanius et al. (2018)

Advancing towards simulations on the biological time scale is a continuous effort. Specialized simulation-dedicated hardware (Shaw et al. 2007; Shaw et al 2014) has enabled tens-to-hundreds microsecond sampling of membrane protein systems. For example, such simulations have provided insight into the energetics of membrane partitioning (Andersson et al. 2013), insertion of outer-membrane proteins (Lundquist et al. 2018), and mechanisms of GPCR receptors (Hollingsworth and Dror 2018), voltage-gated potassium (Jensen et al. 2012) and sodium (Boiteux et al. 2014) channels. Another area of active research is development of enhanced-sampling algorithms. Enhanced sampling methods can enable access to free energy landscapes associated with simulated membrane protein conformational dynamics (Harpole and Delemotte 2018; Howard et al. 2018), and estimate e.g. lipid-binding energetics (Corey et al. 2020). The accelerated weight histogram (AWH) method was used to understand the structural features and thermodynamics underlying ammonia selectivity in aquaporin TIP2;1, a membrane channel permeable to both water and ammonia (Lindahl et al. 2018). A metadynamics approach based on spatial collective variables of a sugar-uptake pathway in the LacY transporter obtained from extended brute-force simulations (Fig. 3a), resulted in a binding free energy that were in excellent agreement with experimental data (Kimanius et al. 2018) (Fig. 3b). Finally, coarse-grained (CG) simulation models enable enhanced sampling by reducing the degrees of freedom in the system, while keeping critical physicochemical properties, although at the cost of atomic resolution (Hedger and Sansom 2016). The CG description has enabled e.g. simulation of multicomponent, asymmetric biological membranes (Marrink et al. 2019) and also containing multiple copies of membrane proteins (Chavent et al. 2016). Despite these impressive advances, simulating membrane proteins in native membranes at the biological time scale of tens-to-hundreds of milliseconds at atomistic detail, does not seem possible within a foreseeable future.

**Fig. 4:**
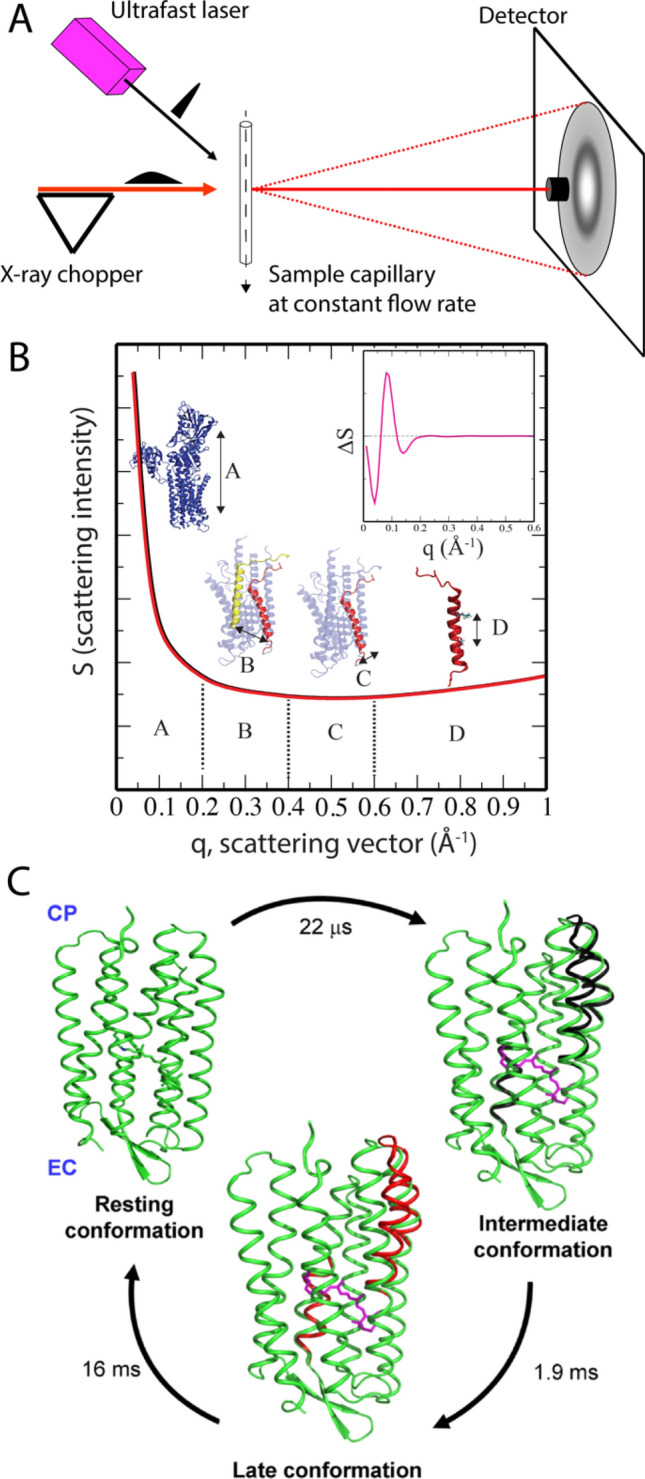
Schematic of the TR-XSS experimental design and resolved membrane protein dynamics. **a** The pump laser pulse arrives at the sample in the capillary (either static or at a continuous flow rate) before the onset of the X-ray probe pulse, which yields concentric rings on the detector. Subtracting non-activated images from the laser-activated images results in difference images that contain the time-resolved structural data. **b** Conventional X-ray scattering profiles from two P-type ATPase protein structures (black and red lines), where q = 4π sin(θ)/λ = 4π/2d, where 1/d is the resolution in X-ray crystallography. E.g. q = 0.2 Å^−1^ corresponds to distances of approximately 30 Å, and q = 1.0 Å^−1^ to approximately 6 Å. An example of a difference TR-XSS spectrum is shown in the inset. **c** Bacteriorhodopsin TR-XSS data identified structures and kinetics of two transient intermediates at 22 μs and 1.9 ms with a subsequent relaxation back to the ground state in 16 ms. The structural changes were about twice those observed in crystal structures and revised the bacteriorhodopsin transport mechanism. Adapted from Andersson et al. (2009)

From a different perspective, time-resolved X-ray solution scattering (TR-XSS) experiments are ideally suited to provide direct X-ray structural fingerprints on the biological micro-to-milliseconds time scale, albeit at low spatial resolution—in a natural membrane environment. Hence, there is temporal and spatial complementarity between the simulation and TR-XSS methodologies and combining the two should, in principle, allow approaching one of the grand challenges in structural biology, namely to observe proteins operate in single turn-over cycles directly in the natural membrane environment. In TR-XSS experiments, a laser pulse triggers the reaction and intense, synchrotron-generated X-ray pulses monitor the structural changes in the liquid sample (Fig. 4a). The methodology developed from studies tracking the time-dependent formation of transient structural intermediates of photoactive chemicals, such as I_2_ (Neutze et al. 2001), CH_2_I_2_ (Davidsson et al. 2005), and C_2_H_4_I_2_ (Ihee et al. 2005), as well as expansion of the surrounding solvent matrix (Georgiou et al. 2006). Because the heavy atoms are strong X-ray scatterers, relatively subtle structural rearrangements result in prominent difference scattering profiles that extend to the wider angles, i.e. contains structural information of higher resolution. The sensitivity of the TR-XSS method was showcased by resolving high-resolution temporal and spatial differences in the dissociation and recombination following photoactivation of CH_2_I_2_ in solvents of varying polarity (Vincent et al. 2009). However, protein large-scale conformational changes involve thousands of atoms and present a very different scenery for TR-XSS visualization. Nevertheless, MD simulations predicted the feasibility of such studies (Andersson et al. 2008).

## Monitoring Conformational Dynamics in Light-Sensitive Proteins

Conventional, i.e. not resolved in time, small-angle and wide-angle X-ray scattering (SAXS/WAXS) experiments result in data that is a rotational and conformational average of the protein structure, observed as concentric rings on the detector. After radial integration, the structural data are represented as scattering intensity as a function of the scattering vector q, where q acts as an atomic ruler (Fig. 4b). At q < 0.2 Å^−1^, the global shape and approximate size of the protein is observed. With widening scattering angles, i.e. at higher q, successively more detail is probed in the protein structure: interactions between internal domains (0.2 < q < 0.4 Å^−1^), interactions between secondary structural elements (0.4 < q < 0.6 Å^−1^), and between individual side chains (q > 0.6 Å^−1^). However, because the protein molecules tumble around in the sample, the high-resolution data is inaccessible. In addition, obtaining a unique structural solution of the 1-dimensional scattering spectrum, poses an immense challenge. TR-XSS experiments monitor only the structural differences in the sample, and hence reduce the complexity of the data enormously. Also, the experimental data contain information of the structural changes of all the atoms in the protein molecule, in contrast to traditional spectroscopic methods that register the immediate environment around a certain probe. To obtain the difference scattering profile, a spectrum obtained without laser excitation is subtracted from that registered at a particular point in time after the laser flash. On the micro-to-milliseconds time scale, the obtained difference spectra suffer from low signal-to-noise ratio. Therefore, the TR-XSS experiment needs to be repeated typically hundred to thousands of times, which sets demands on sample size.

Another important prerequisite for a successful TR-XSS experiment is that a large enough population of the species to be studied needs to be activated simultaneously. In this way, synchronized structural dynamics will be performed as the reaction propagates, which directly affects the experimental signal-to-noise. Fast-mixing devices can trigger e.g. protein folding/unfolding (Akiyama et al. 2002), and laser activation can provide an exact trigger—given that the reaction is photosensitive. In the first TR-XSS protein experiment, conformational changes of human hemoglobin were resolved at nanosecond time resolution (Cammarata et al. 2008). Laser-induced release of bound carbon monoxide was used to trigger the reaction, which then was followed from 200 ns to 32 ms. The protein was observed to form the ‘tense’ structure, which is stable in the absence of the ligand, in less than 100 μs. Light-sensitive proteins carry an inherent light trigger and are hence prime candidates for TR-XSS characterization. Such experiments have increased understanding of how bacterial sensor histidine kinases induce signaling networks in bacteria (Takala et al. 2014; Berntsson 2017). In particular, local structural rearrangements were resolved on the μs timescale in the vicinity of the chromophore with a subsequent rotational conformational change occurring within a few milliseconds (Björling 2016).

The first TR-XSS study of a membrane protein focused, not surprisingly, on archaeal rhodopsins including the proton transporter bacteriorhodopsin, well-known for its stability (Andersson et al. 2009). Structural modeling of the time-resolved data involved rigid-body movements based on morphing trajectories extracted from a plethora of available bacteriorhodopsin crystal structures trapped in different states of the photocycle. The resulting structures of transient states at 22 microseconds and 1.9 milliseconds showed that the laser-triggered isomerization of the retinal cofactor induced helical rearrangements that increased over time (Fig. 4c). Helical movements were present already in advance of the so-called primary proton transfer at the retinal, which was in agreement with trapped crystal structure intermediates showing reorganization of the internal H-bond network (Edman et al. 2004; Neutze et al. 2002; Royant et al. 2000). The observed conformational changes in the detergent-micelle solution were about twice the magnitude of those observed in protein crystals, which showcases the restriction posed by crystal lattices. Following the proof-of-principle membrane protein TR-XSS study, conformational changes have also been recorded for proteorhodopsin in detergent-micelles (Malmerberg et al. 2011) and bovine rhodopsin in native rod disc membranes from the bovine retina (Malmerberg et al. 2015).

## Indirect Activation of Membrane Protein Dynamics

**Fig. 5:**
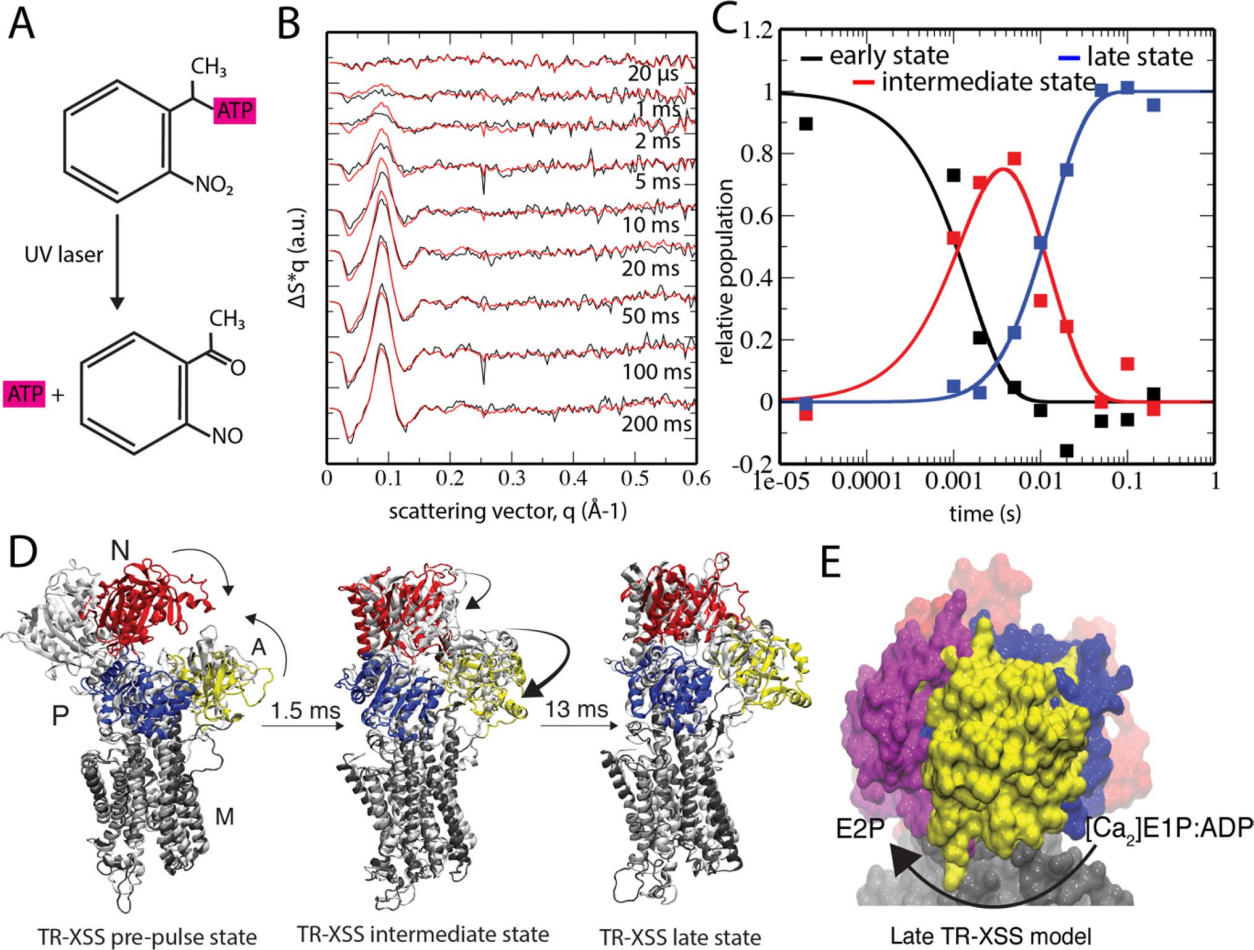
TR-XSS characterization of Ca^2+^ ATPase (SERCA) kinetics and structural dynamics. **a** The chemistry of laser-induced release of ATP from caged ATP. **b** TR-XSS data of SERCA in native membranes (black). The red lines display reconstituted data according to the best-fitting kinetic model. **c** Temporal shifts in the population densities of the identified transient states; early (black), intermediate (red), and late (blue). **d** Refined TR-XSS models of prepulse, intermediate, and late states are shown with the closest corresponding crystal structure (white) and rise times from the kinetic analysis. **e** Structural differences in the A domain between the late TR-XSS model (yellow), the preceding [Ca_2_]E1P:ADP crystal structure (blue), and the subsequent E2P crystal structure (magenta). The N and M domains are colored red and gray, respectively. The arrow indicates the forward reaction. Adapted from Ravishankar et al. (2020)

Transition of the TR-XSS methodology to include membrane proteins not inherently sensitive to light requires finding an indirect means of activating the protein. Caged compounds containing photoremovable groups provide control over release of e.g. ions, neurotransmitter, and ATP molecules (Klan et al. 2013) —and constitutes a possible indirect trigger of protein activity (Fig. 5a). Light-induced ATP release from caged ATP has been used to monitor P-type ATPase activity and time-dependent evolution of structural features with Fourier-Transform Infra-Red (FTIR) spectroscopy (Barth et al. 1996; Ravishankar et al. 2018). Caged ATP activation was also used in early grazing X-ray incidence diffraction studies to trigger synchronization of SERCA reaction cycles and monitoring of intermediate states in partially dehydrated multilayers (Blasie et al. 1985). However, such lamellar diffraction experiments can only resolve profile differences perpendicular to the membrane normal, while solution-scattering studies in principle enable structural determination of 3-dimensional protein envelopes. Nevertheless, the early X-ray studies further showed feasibility for caged ATP triggering of P-type ATPase transport dynamics. Because caged compound activation is an irreversible reaction, the protein solution needs to flow continuously across the focal spots of the laser and X-rays, which sets immense demands on the experimental setup and access to large quantities of the membrane protein.

**Fig. 6:**
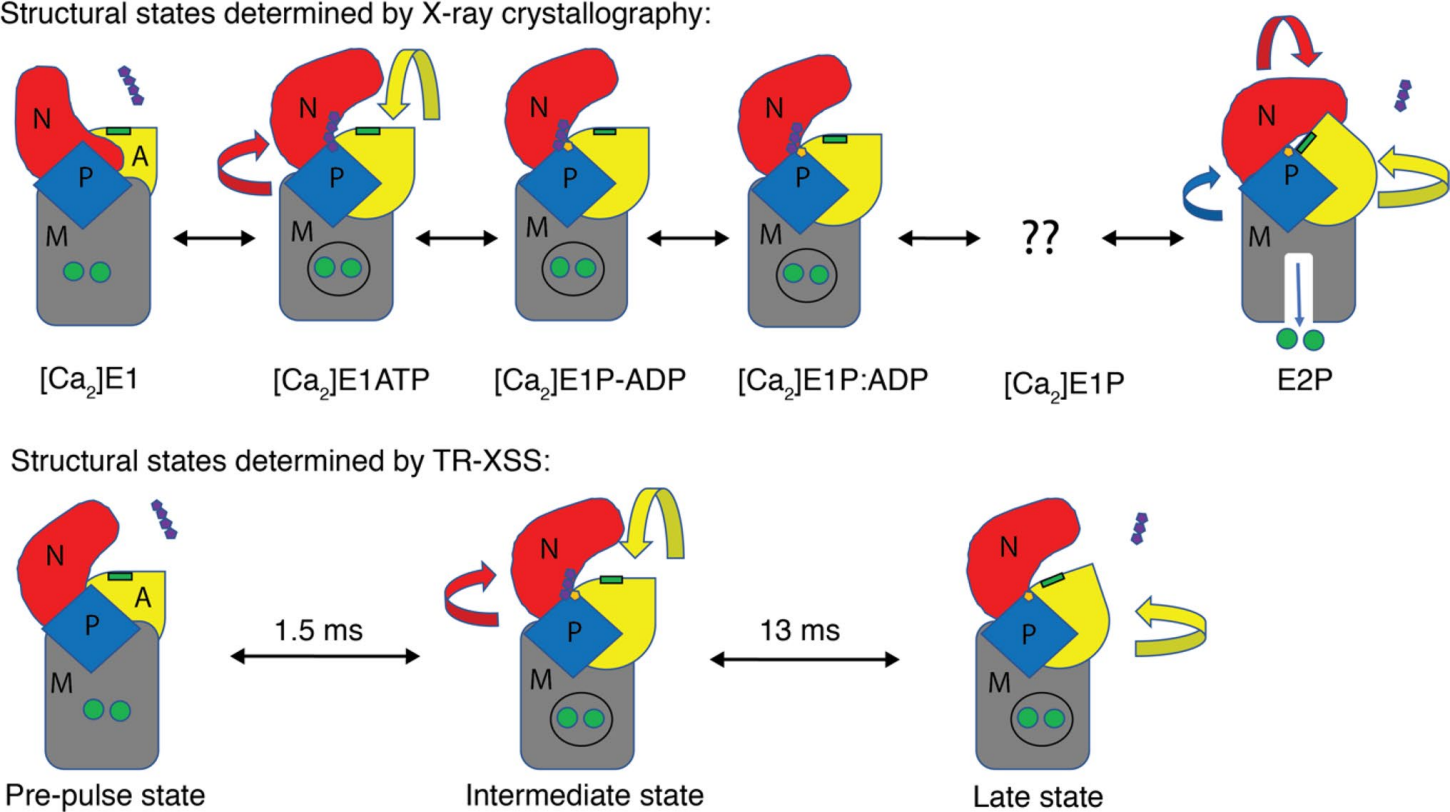
Schematic comparison of the principal structural rearrangements in-between crystal structures and TR-XSS models. The pre-pulse state shows reduced opening of the cytoplasmic domains, the intermediate TR-XSS model is similar to a [Ca_2_]E1ATP state, and displacement of the A domain in the late TR-XSS model exposes the ADP site, but with the N-domain not yet in a E2 position. The ATP and ADP displayed as four and three purple pentagons, the TGES motif is represented by a green rectangle, and phosphorylated aspartic acid in yellow, and the calcium ions are depicted as green circles. Adapted from Ravishankar et al. (2020)

In the first TR-XSS experiment on a non-light sensitive membrane protein, structural dynamics were recorded in sarcoplasmic reticulum (SR) membranes from rabbit skeletal muscle from 20 μs to 200 ms following laser-activation of caged ATP (Ravishankar et al. 2020) (Fig. 5b). The protein content in SR membranes consist to 90% of the SERCA protein (Meissner et al. 1973), which enabled monitoring activity directly in the native membrane. The time-resolved data contained structural fingerprints of two transient intermediate states, at 1.5 ms and 13 ms, respectively (Fig. 5c). Development of a structural refinement protocol based on MD simulation enabled structural interpretation of the identified transient states. In this way, the 1.5-ms intermediate was found to represent a calcium-bound state where the cytoplasmic domains had closed upon the ATP substrate (Fig. 5d). Such domain movements had also been observed in X-ray crystallography (Dyla et al. 2019). In contrast, refinement of the 13-ms intermediate showed a novel arrangement of the cytoplasmic domains (Fig. 5d). In this state, the ADP-binding site was exposed and the so-called actuator (A) domain, which dictates membrane opening and closing, was positioned in-between the principal locations that determine whether the transporter is opened towards the cytoplasm or the SR lumen (Fig. 5e). The existence of such a state had been inferred from biochemical (Danko et al. 2009) and fluorescence microscopy data (Dyla et al. 2017). Hence, the TR-XSS study presented new structural information and kinetics for the decisive “moment of truth” intermediate of SERCA inward-to-outward transport in a native membrane (Fig. 6). This work highlights TR-XSS in combination with MD simulation as a powerful tool to determine structures of transient, high-energy intermediates of membrane proteins in complex lipid settings. The structural refinement relies on existence of high-resolution data, and the TR-XSS methodology should therefore be viewed as a complementary structural-biology technique that is highly timely since it capitalizes on advances in e.g. cryogenic electron microscopy (cryo-EM).

## Future Perspective

Identification of structural dynamics of the protein and surrounding membrane is key to understanding membrane protein function. While the TR-XSS methodology holds great promise to contribute to the membrane protein field, the technique is far from standardized and much developmental work is required. For instance, the proof-of-principle TR-XSS characterization of a membrane protein using indirect activation targeted a P-type ATPase protein (Ravishankar et al. 2020). These transporters are exceptional in that the cytoplasmic domains in P-type ATPases protrude significantly from membrane and undergo large-scale conformational change. Therefore, it was possible to model the X-ray scattering data without accounting for dynamics in the membrane. To pave way for similar characterization of less pronounced structural changes in proteins lacking protruding domains, strategies to handle contributions from the surrounding lipids need to be developed. In fact, this presents an opportunity to explore the allosteric nature of membrane lipids. Given the developments within the field of caged-compound chemistry (Klan et al. 2013), electric-field-stimulated protein dynamics in time-resolved crystallography (Hekstra et al. 2016), and synthetic photoswitches (Gorostiza and Isacoff 2008), several possible target proteins can now be envisioned. Also, advances of X-ray free-electron lasers (XFELs) delivering extremely brilliant X-ray pulses have provided access to picosecond structural dynamics, exemplified by myoglobin (Levantino et al. 2015) and a bacterial photosynthetic reaction center (Arnlund 2014). Finally, MD simulations can enable refinement of TR-XSS data. However, such approaches have so-far been case-specific and can potentially suffer from limited sampling, in particular of dynamics of proteins inserted into complex lipid bilayers. While the SERCA MD-based refinement protocol utilized enhanced sampling (Ravishankar et al. 2020), more sophisticated methods have been suggested. For example, energetic restraints can enable driving simulations toward agreement with experimental data using a harmonic biasing potential (Chen and Hub 2015; Björling et al. 2015) or a metadynamics collective variable (Kimanius et al. 2015). Despite the challenges, TR-XSS characterization has emerged as a powerful tool in structural biology to probe protein and lipid dynamics and holds great potential to contribute to understanding of membrane protein functioning and associated disease.
